# Bone Morphogenetic Protein Receptor 2 (BMPR2)/TAK1-d signaling regulates the phosphorylation of Ser76 in MCUb to control mitochondrial Ca2+ levels and survival of cancer cells

**DOI:** 10.21203/rs.3.rs-10486044/v1

**Published:** 2026-10-21

**Authors:** Manohar Singh, Hellen chiou, Ashley Toussaint, Dongxuan Jia, John Gilleran, Jacques Roberge, Daniel Herranz, John Langenfeld

## Abstract

Background Inhibiting bone morphogenetic protein receptor 2 (BMPR2) signaling downregulates TAK1-d that increases mitochondrial Ca ^2+^ (mtCa ^2+^ ) levels. BMPR2 inhibition in non-small cell lung carcinoma (NSCLC) cells and mouse hippocampal cells increases mitochondrial bioenergetics. In leukemia cells, BMPR2 inhibition causes more cell death, higher mtCa ^2+^ levels and less ATP production compared to NSLC. In this study, we examined whether BMPR2/TAK1-d signaling phosphorylates Ser92 in the mitochondrial uniporter (MCU) and/or Ser76 in MCUb, a dominant-negative MCU paralog, to control mtCa ^2+^ levels. In addition, we explored whether combining a BMPR2 inhibitor with a BCL-2 inhibitor induces mtCa ^2+^ overload and cell death. Furthermore, the effects of phosphorylating Ser76 MCUb on ATP levels was studied. Methods mtCa ^2+^ and mitochondrial superoxide anion (mtROS) levels were assessed with Rhod-2AM and MitoSox Red staining respectively and quantified by flow cytometry. BMPR2 inhibitor JL189, TAK1-d inhibitor (5Z-7-Oxozeaenol), siRNA targeting BMPR2, TAK1, MCU, and MCUb, cDNA overexpression, and genetic deletions were used to determine whether BMPR2/TAK1-d signaling phosphorylated Ser92 in MCU and/or Ser76 in MCUb. MCUb knockout cells reconstituted with wild-type MCUb, kinase inactive Ser76/A MCUb, or constitutively active Ser76/E MCUb were used to assess the effects of the phosphorylation of Ser76 MCUb on mtCa ^2+^ levels. CellTiter Glo was used to determine ATP levels. Results JL189 combined with BCL-2 inhibitors synergistically induces cell death of NSLC and leukemia cells associated with a synergistic increase in mtROS and mtCa ^2+^ levels. Inhibiting BMPR2/TAK1-d signaling enhances mtCa ^2+^ influx induced by ROS. BMPR2/TAK1-d signaling phosphorylates both Ser76 in MCUb and Ser92 in MCU. Phosphorylation of Ser76 MCUb decreases mtCa ^2+^ levels and ATP levels, while decreasing the phosphorylation of Ser76 MCUb increases mtCa ^2+^ levels. Importantly, chemotherapy resistant p53 mutated acute myeloid cells leukemia are highly sensitive to the combination of JL189 and venetoclax. Conclusion Our studies suggest that inhibiting BMPR2/TAK1-d signally promotes synergistic cell death when combined with BCL-2 inhibitors and other therapeutics by inducing mtCa ^2+^ overload which is mediated in part, by decreasing the phosphorylation of Ser76 MCUb. Furthermore, our studies suggest that BMPR2/TAK1-d regulation of Ser76 in MCUb are pleotropic, regulating both cell survival and mitochondrial bioenergetics.

